# Photobiomodulation therapy combined with static magnetic field in tibial fracture healing of a dog: A case report

**DOI:** 10.1002/vms3.1071

**Published:** 2023-01-14

**Authors:** Iker M. Asteinza Castro, Armando Amador Morga, Douglas Scott Johnson

**Affiliations:** ^1^ Division of Ortophedic Care Animal Home Veterinary Hospital Mexico City Mexico; ^2^ Research and Development Multi Radiance Medical Solon Ohio USA

**Keywords:** bone healing, dog, light‐emitting diodes, low‐level laser therapy, photobiomodulation, surgery, tibia fracture

## Abstract

A 10‐week‐old male, Xoloitzcuintle (Mexican hairless dog), weighing 8.9 kg was presented after its owner accidentally stepped on its paw. The dog presented with acute pain, inflammation and grade IV lameness in the right hind paw. A complete transverse fracture in the right proximal tibia was diagnosed from radiography. The dog underwent a minimally invasive plate osteosynthesis (MIPO) procedure. After surgery, photobiomodulation therapy combined with static magnetic field (PBMT‐sMF) was applied twice daily for 21 days. A multi‐wavelength PBMT‐sMF device was applied at three sites using different frequencies: proximal and distal of the fracture zone (3000 Hz, 40.35 J per site, and 300 s per site) and in the fracture zone (250 Hz, 39.11 J and 300 s per site). Follow up radiographies were performed after surgery and treatment with PBMT‐sMF. Eighteen days post‐surgery the healing process of bone was advanced. Fifty‐five days post‐surgery the callus was enlarged. In addition, radiographic union and clinical union was evidenced by closure of the fracture gap. This case report has reported the use of PBMT‐sMF in order to accelerate and improve bone healing following a MIPO procedure on a complete transverse fracture in the proximal tibia of a puppy.

## Graphical abstract

‐ PBMT‐sMF can accelerate bone healing after a fracture in proximal tibia of puppy.
‐ PBMT‐sMF can improve bone healing after a fracture in proximal tibia of puppy.
‐ The addition of PBMT‐sMF to the MIPO procedure may be an efficient treatment after bone fracture.

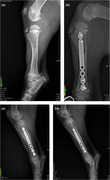

## 1 INTRODUCTION

Fractures in tibia are very common in small animals, being the third most common fracture type and representing 20% of long bone fractures (Gorse, 1998). Long bone fracture usually triggers other signs and symptoms such as limb dysfunction, pain, instability, overlying soft tissue injured, abnormal limb posture or crepitus (Roush, 2005). The location and type of fracture may vary between skeletally immature animals and skeletally mature animals. For instance, proximal tibial fractures are usually observed only in very young animals, skeletally immature (Zaal & Hazewinkel, 1996; Johnson & Boone, 1993).

The appropriate treatment of tibial fractures must be determined after taking into consideration mechanical and biological factors and patient adherence (Palmer, 1999). Over the years, minimally invasive techniques have been used to repair tibial fractures (Schmökel et al., 2007; Schmokel et al., 2003). This type of technique preserves blood supply, encourages faster healing, reduces morbidity and accelerates functional recovery (Schmökel et al., 2007; Schmokel et al., 2003). Among the surgical approaches it could be ‘open but don´t touch’ (OBDT) approach, minimally invasive surgery (MIS) (Schmökel et al., 2007; Schmokel et al., 2003) and minimally invasive percutaneous plate osteosynthesis (MIPO) (Pozzi & Lewis, 2009). MIPO can use two types of bone‐plating systems: traditional bone plates and locking plates (Schwarz, 2005). MIPO may have an advantage over other approaches since it reduces surgical time, and consequently reduces the risk of infection (Hudson et al., 2009).

There are several methods that can be used to improve bone healing after fracture, including the use of a therapeutic intervention called photobiomodulation therapy (PBMT) applied alone or combined with static magnetic field (PBMT‐sMF). Photobiomodulation therapy is a non‐thermal process applied in the form of light amplification by stimulated emission of radiation (LASER) and light‐emitting diodes (LEDs) (Anders et al., 2015). The light emitted by PBMT (and PBMT‐sMF) interacts with photoreceptors present in mitochondria triggering stimulation or inhibition of cellular metabolism in different tissues (Anders et al., 2015; Karu, 1989).

In recent years, the number of in vitro, preclinical and clinical studies in humans investigating the effects of PBMT has increased. However, there is still a scarcity of quality studies observing and investigating the effects of PBMT in the veterinary field. To date, was observed that the use of PBMT is associated with increased peak vertical force after tibial plateau levelling osteotomy (Rogatko et al., 2017) and improvement of function after surgical correction of a herniated disc in dogs (Draper et al., 2012). In addition, it was observed that PBMT may accelerate chronic wound healing process in dogs (Hoisang et al., 2021) and reduce lameness, pain and non‐steroidal anti‐inflammatory drugs (NSAIDs) use on canine osteoarthritis (Looney et al., 2018). Finally, there is evidence that PBMT has a potential as a biostimulator, inducing bone formation during distraction osteogenesis in dogs, resulting in shorter treatment time and improved quality and quantity of new bone (Taha et al., 2018). To date, the effects of PBMT on bone healing in dogs have not been investigated, however, in vitro and preclinical studies showed that PBMT increases vascularisation and modulates inflammatory response, enhancing the bone matrix synthesis and neoformation (Pinheiro & Gerbi, 2006), and increasing fibroblast growth factors (Saygun et al., 2008), osteocytes (Dörtbudak et al., 2002), osteoblastic proliferation (Pinheiro & Gerbi, 2006) and collagen deposition (Pinheiro & Gerbi, 2006). In addition, there is evidence that the sooner the treatment with PBMT starts, when high cellular proliferation occurs, the more effective it will be (Pinheiro & Gerbi, 2006).

In the case described herein, we aim to report the use of PBMT‐sMF after a MIPO procedure in a puppy with complete transverse fracture in the right proximal tibia.

## 2 CASE REPORT

**FIGURE 1:**
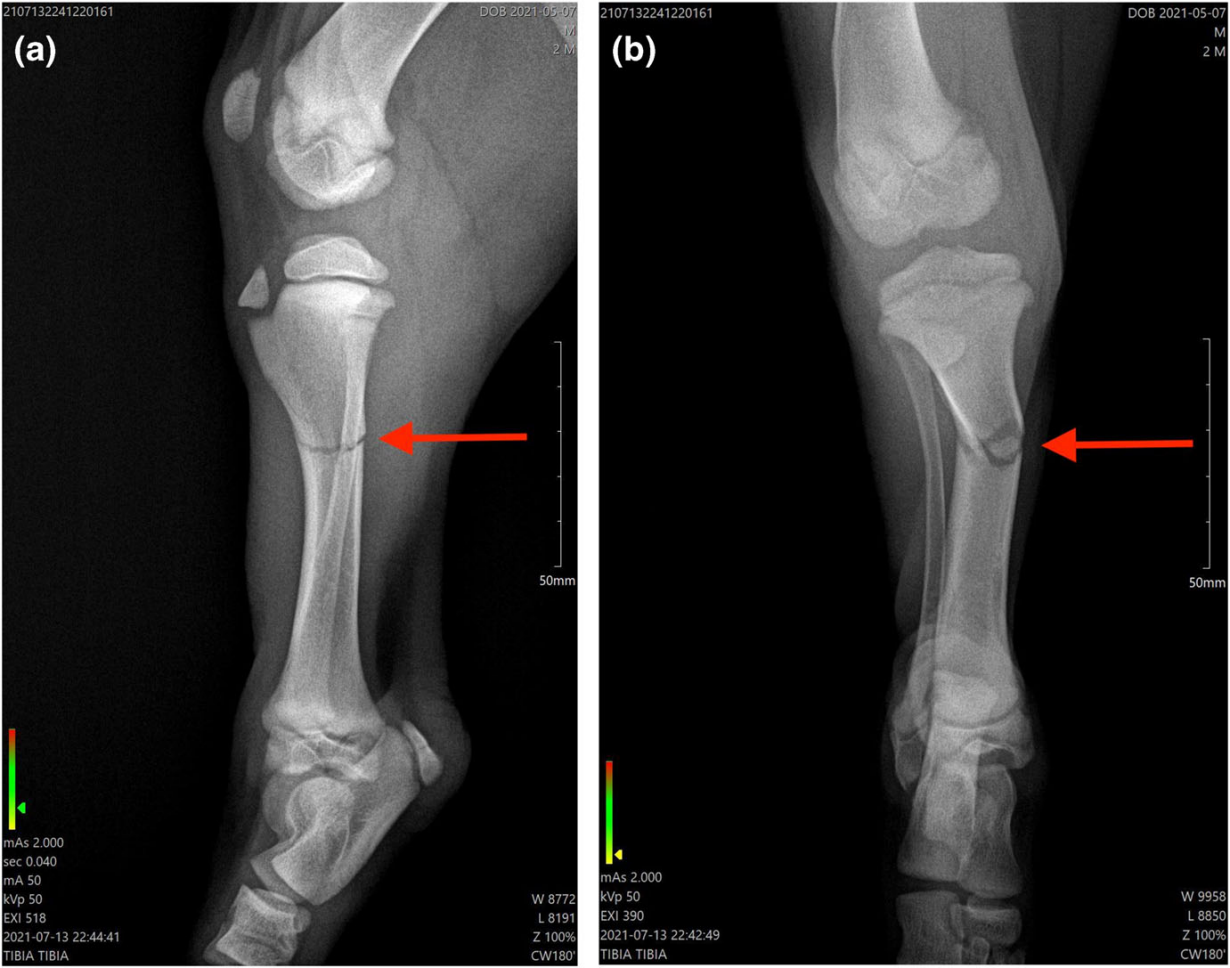
Radiography before procedures. (a) Radiography in mediolateral view before surgery and treatment with PBMT‐sMF. The arrow demonstrates a transverse fracture in proximal tibia. (b) Radiography in craniocaudal craneo caudal view before surgery and treatment with PBMT‐sMF. The arrow demonstrates a transverse fracture in proximal tibia. The figure is owned by the authors.

A male Xoloitzcuintle dog, 10 weeks old, weighing 8.9 kg was presented to the Animal Home Veterinary Hospital in Mexico City, Mexico Veterina, after its owner accidentally stepped on its paw. The dog presented a normal clinical examination. However, the orthopaedic exam revealed acute pain, inflammation (swelling, heat and loss of function) and grade IV lameness (non–weight‐bearing lameness) in the right hind paw. A lameness score from 0 (no lameness) to 5 (no ambulatory) was used to measure the lameness grading (Impellizeri et al., 2000; Drygas et al., 2011). A radiography showed a complete transverse fracture in the right proximal tibia, type 43 (Figure 1) (Johnson et al., 2005).

The dog´s owner was concerned about the fracture healing because the puppy, skeletally immature, is a show prospect coming from international champions of the breed. Thus, both function and aesthetic aspects would be important. The owner was also concerned that inadequate fracture healing could affect locomotion and gait.

**FIGURE 2:**
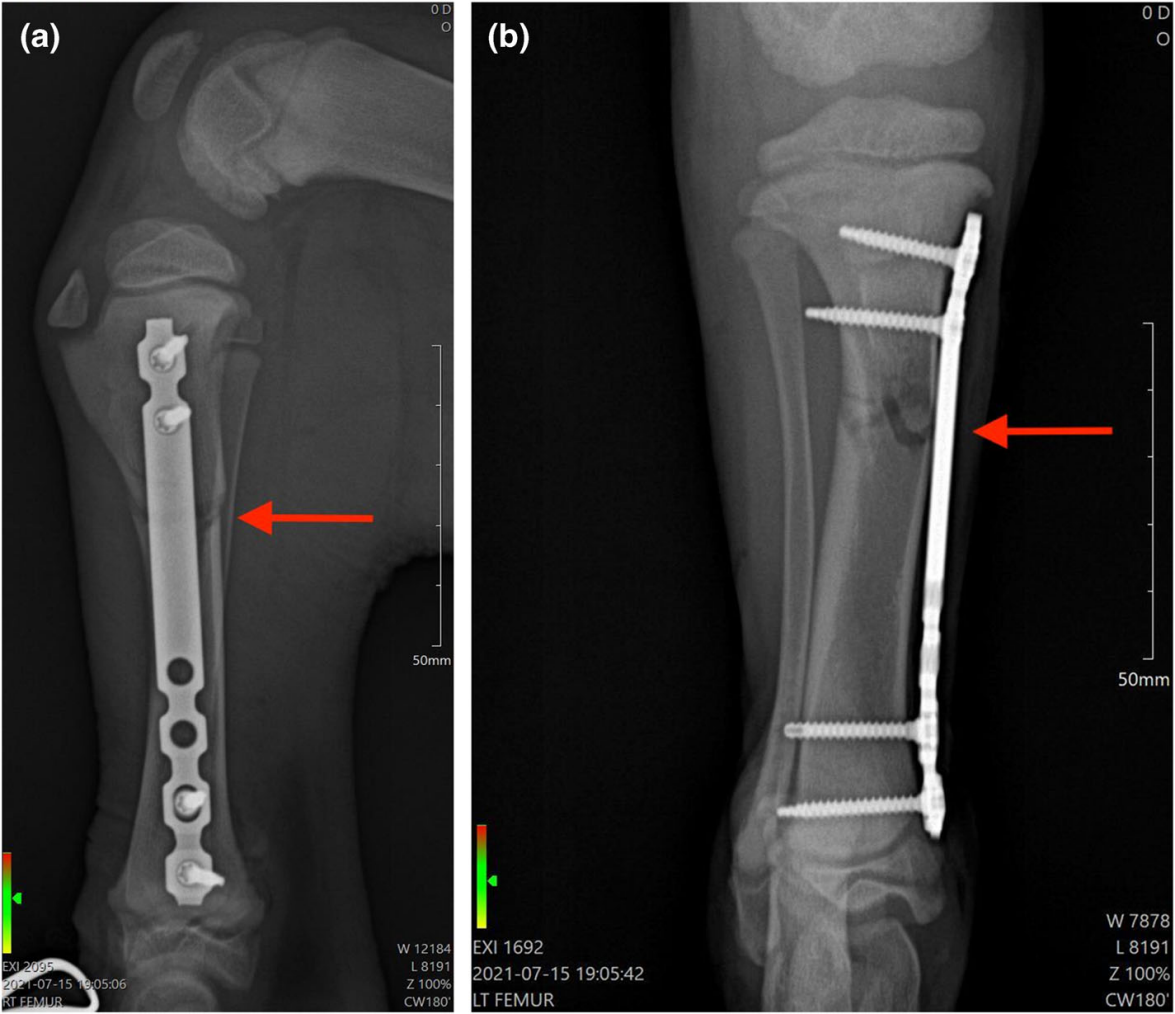
Radiography post‐surgery. (a) Radiography in mediolateral view post‐surgery and before treatment with PBMT‐sMF. The arrow demonstrates a transverse fracture in proximal tibia. (b) Radiography in craniocaudal view post‐surgery and before treatment with PBMT‐sMF. The arrow demonstrates a transverse fracture in proximal tibia. In the immediate post‐surgery radiographic study, in its craneo caudal projection, we can observe the presence of a radiopaque implant on the medial aspect of the tibia from the proximal metaphysis to the distal metaphysis, with the presence of 2 proximal and 2 distal locking screws. The locking screw number 1 is placed monocortically and locking screws 2, 3 and 4 are placed bicortically. The figure is owned by the authors.

At first, the main focus of treatment was pain control and fracture stabilisation until surgery could be scheduled. The fracture stabilisation was done using a Robert‐Jones bandage (RJB), in order to increase patient comfort, to minimise local soft tissue swelling and to prevent additional soft tissue injury (Roush, 2005). It was prescribed oral carprofen 2.2 mg/kg twice a day, oral omeprazole 1 mg/kg once a day and oral gabapentin 10 mg/kg three times a day (Plumb, 2005). Two days later, the dog underwent to surgery. The protocol for general anaesthesia was as follow: water fasting of 2 h and food restriction of 8 h before the procedure. It was used acepromazine 0.05 mg/kg and meloxicam 0.2 mg/kg as premedication. It was used propofol 2–5 mg/kg intravenous to cause the effective loss of the laryngotracheal reflex. Anaesthetic maintenance was performed with sevoflurane vaporised in oxygen (O2), using a rebreathing circuit. The vaporiser setting was adjusted to maintain a surgical‐anaesthetic plane based on the eye position, mandibular tonus, and absence of autonomic reflex to nociceptive stimulus (Plumb, 2005). MIPO procedure, with titanium locking compression plate (OsTi‐Lok, OsteoCertus, FL, USA), with two screws fixed distally and proximally was performed. This approach and this plate/screw configuration was chosen in order to promote healing and minimise infection risks. The function of a locking plate is as an internally placed external fixator, favouring an environment for secondary bone healing via callus formation through relative stability by maintaining fracture gap strains under 10% (Schwarz, 2005). For bridging fixation, two to four screws either side of the fracture gap is recommended (Schwarz, 2005). In these applications, increasing the number of the screws in the construct does not necessarily equate to an increased construct stability (Schwarz, 2005). In this specific case, MIPO was chosen because it preserves periosteal blood supply compared to open plating, which may accelerate bone healing (Schütz & Südkamp, 2003). Post‐surgery, another radiography was performed (Figure 2).

### 2.1 Post‐surgery intervention

**FIGURE 3:**
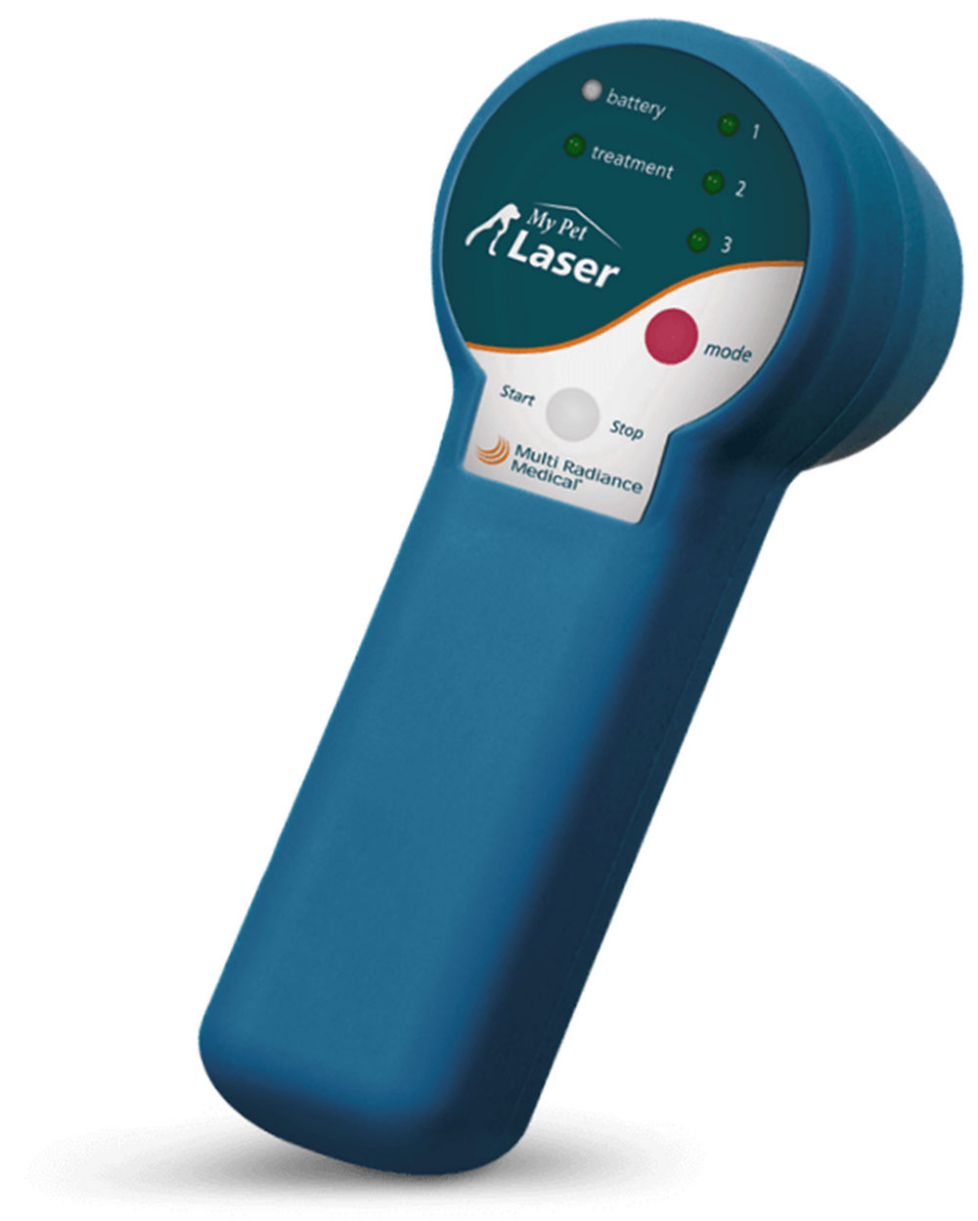
Cordless and portable My Pet Laser™ (MPL) device.

For 21 days, oral omeprazole 1 mg/kg once a day, oral carprofen 2.2 mg/kg twice a day, and oral gabapentin 10 mg/kg three times a day were prescribed (Plumb, 2005). In addition, PBMT‐sMF was applied for 21 days, twice per day. PBMT‐sMF was applied using a cordless, portable My Pet Laser™ (MPL) device manufactured by Multi Radiance Medical, Solon, Ohio, USA (Figure 3). The multi‐wavelength PBMT‐sMF was applied at three sites with different frequencies: proximal and distal of the fracture zone (MPL 3000 Hz) and in the fracture zone (MPL 250 Hz). Proximal and distal of the fracture zone, PBMT‐sMF was applied using the MPL cluster emitter containing the following 9 diodes and settings: 1 super‐pulsed laser diode (905 nm, 3000 Hz frequency, 4.50 mW average power, 15 W peak power, and 1.35 J dose for each diode), 4 red LEDs (640 nm, 15 mW average power, and 4.5 J dose for each diode) and 4 infrared LEDs (875 nm, 17.5 mW average power and 5.25 J dose for each diode). The total dose applied per site was 40.35 J. The irradiation time per site was 300 sec. In the fracture zone PBMT‐sMF was applied using the MPL cluster emitter containing the following 9 diodes and settings: 1 super‐pulsed laser diode (905 nm, 250 Hz frequency, 0.375 mW average power, 15 W peak power, and 0.1125 J dose for each diode), 4 red LEDs (640 nm, 15 mW average power, and 4.5 J dose for each diode) and 4 infrared LEDs (875 nm, 17.5 mW average power, and 5.25 J dose for each diode). The total dose applied per site was 39.11 J. The irradiation time per site was 300 s. The full description of PBMT‐sMF parameters is provided in Table 1.

**TABLE 1:** Parameters for MyPetLaser™ device

Number of infrared super‐pulsed lasers	1	1
Wavelength (nm)n	905 (±1)	905 (±1)
Frequency (Hz)	3000	250
Peak power (W) – each	15	15
Average mean optical output (mW) – each	4.50	0.375
Power density (mW/cm^2^) – each	10.23	0.852
Energy density (J/cm^2^) – each	3.069	0.2556
Dose (J) – each	1.35	0.1125
Spot size of laser (cm^2^) – each	0.44	0.44
Number of red LEDs	4	4
Wavelength of red LEDs (nm)	640 (±10)	640 (±10)
Frequency (Hz)	2	2
Average optical output (mW) – each	15	15
Power density (mW/cm^2^) – each	16.67	16.67
Energy density (J/cm^2^) – each	5	5
Dose (J) – each	4.5	4.5
Spot size of red LED (cm^2^) – each	0.9	0.9
Number of infrared LEDs	4	4
Wavelength of infrared LEDs (nm)	875 (±10)	875 (±10)
Frequency (Hz)	16	16
Average optical output (mW) – each	17.5	17.5
Power density (mW/cm^2^) – each	19.44	19.44
Energy density (J/cm^2^) – each	5.83	5.83
Dose (J) – each	5.25	5.25
Spot Size of LED (cm^2^) – each	0.9	0.9
Magnetic field (mT)	35	35
Irradiation time per site (s)	300	300
Total dose per site (J)	40.35	39.11
Aperture of device (cm^2^)	4	4
Application mode	Scanning cluster probe in skin contact with a 90° angle and slight pressure	Cluster probe held stationary in skin contact with a 90° angle and slight pressure

### 2.2 Post‐treatment outcomes

**FIGURE 4:**
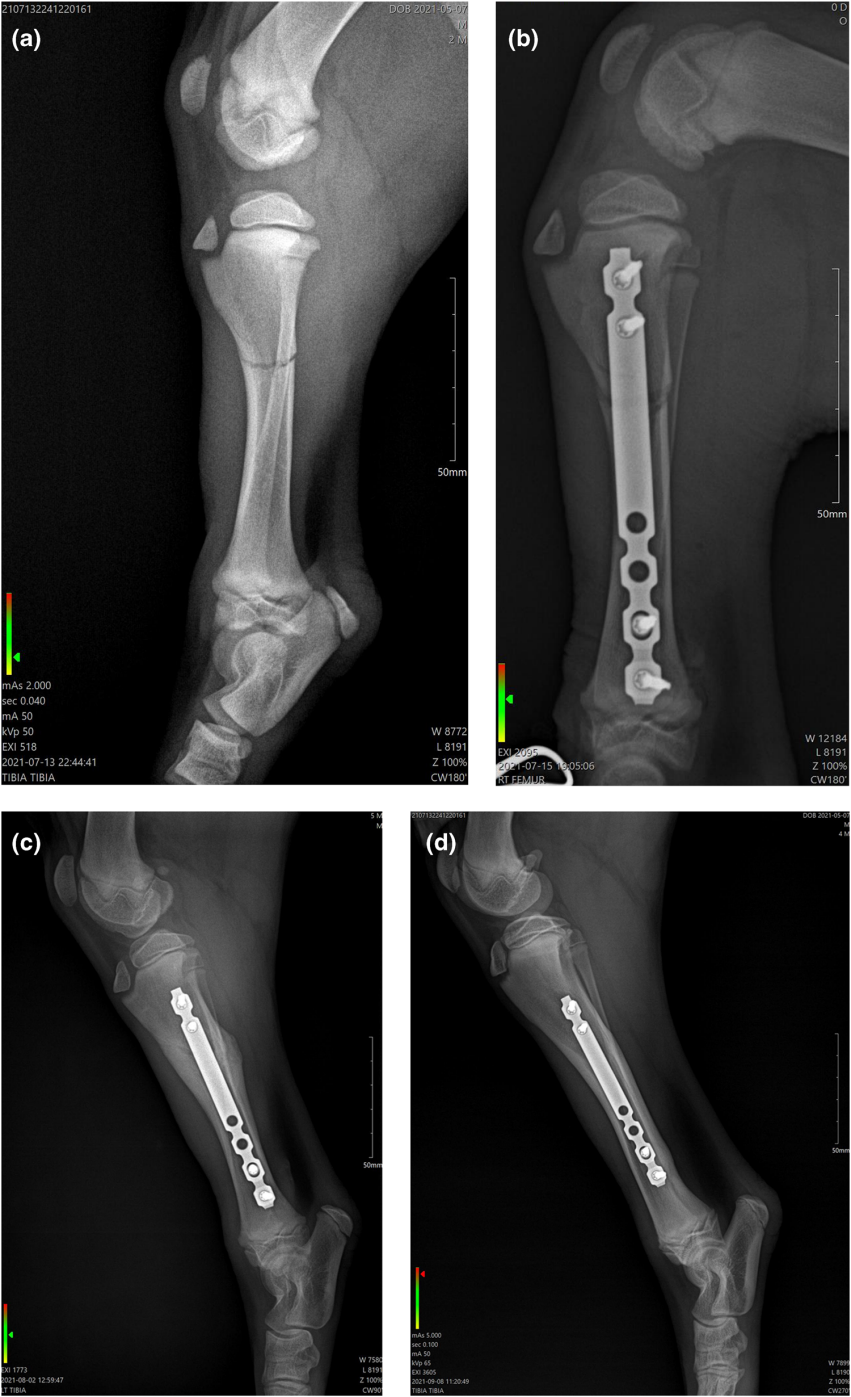
Progression of osteosynthesis using MIPO and PBMT‐sMF in mediolateral view. (a) Radiography before surgery and treatment with PBMT‐sMF shows a simple‐diaphyseal fracture. (b) Radiography immediately post‐surgery and before treatment with PBMT‐sMF shows a transverse fracture in proximal tibia and the fixation with locked titanium plate, with two screws fixed distally and proximally. (c) Radiography at 18 days post‐surgery shows an important periostic reaction suggestive formation of bone callus. (d) Radiography at 55 days post‐surgery shows callus enlarged with bone remodelling. The figure is owned by the authors.

**FIGURE 5:**
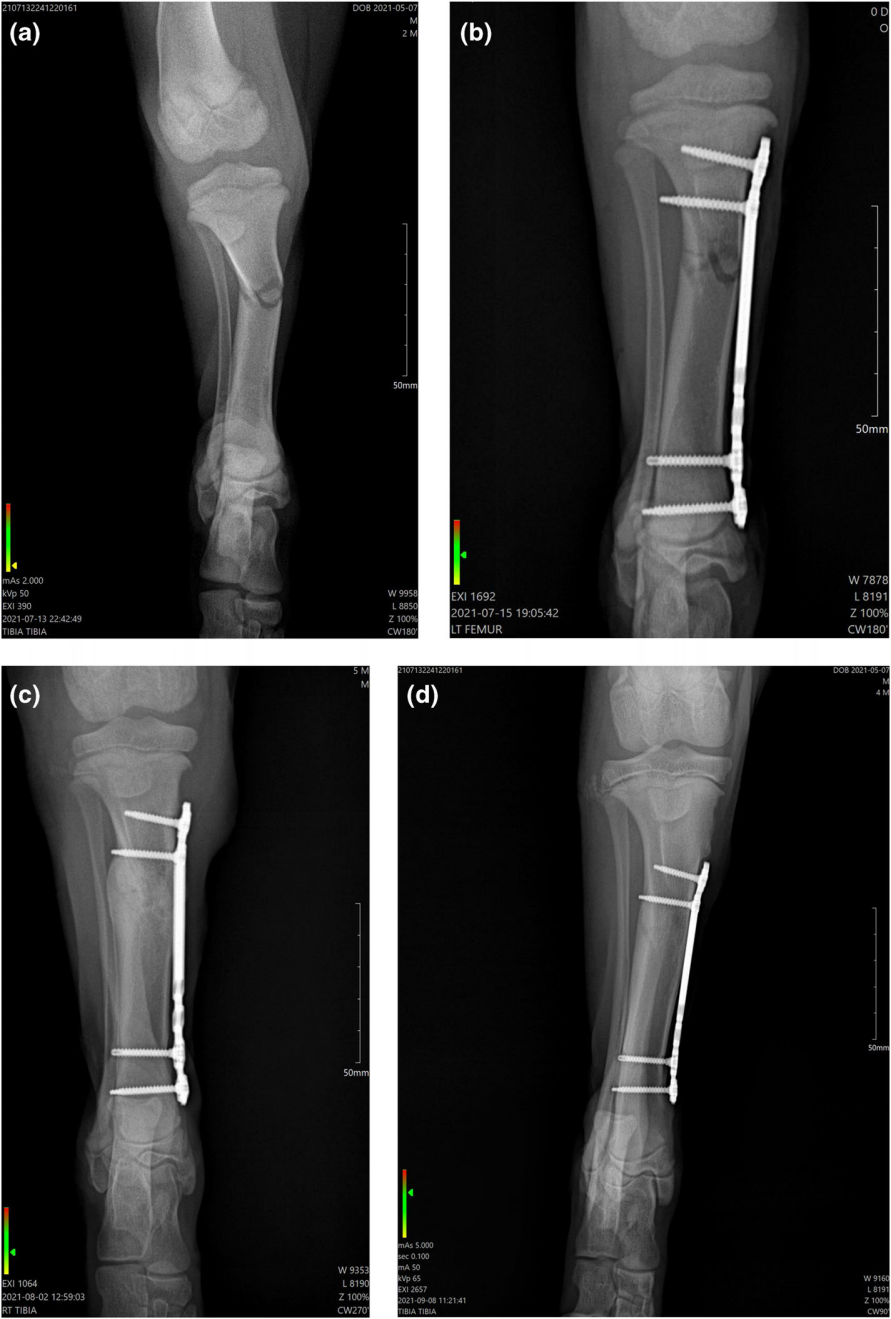
Comparison between radiographies in craniocaudal view. (a) Radiography before surgery and treatment with PBMT‐sMF shows a transverse fracture in proximal tibia. (b) Radiography immediately post‐surgery and before treatment with PBMT‐sMF shows a transverse fracture in proximal tibia and the fixation with locked titanium plate, with two screws fixed distally and proximally. (c) Radiography at 18 days post‐surgery shows an important periostic reaction suggestive formation of bone callus. (d) Radiography at 55 days post‐surgery shows callus enlarged with bone remodelling. The figure is owned by the authors.

Follow up radiographies were performed 18 days post‐surgery (and treatment with PBMT‐sMF) and 55 days post‐surgery (34 days after the end of treatment with PBMT‐sMF). Eighteen days post‐surgery, radiography revealed that although there was still a visible fracture line, the healing process of bone was advanced. Fifty‐five days post‐surgery, radiography revealed that the callus was enlarged, still characterising a remodelling phase. In addition, radiographic union and clinical union was evidenced by closure of the fracture gap. Figure 4 shows the comparison between radiographies in anteroposterior view: before surgery and treatment, day 0 (post‐surgery and before treatment), day 18 (18 days post‐surgery) and day 55 (55 days post‐surgery). Figure 5 shows the comparison between radiographies in lateral view: before surgery and treatment, day 0 (post‐surgery and before treatment), day 18 (18 days post‐surgery) and day 55 (55 days post‐surgery).

## 3 DISCUSSION

This study reported the use of PBMT‐sMF after a MIPO procedure in a puppy with complete transverse fracture in the right proximal tibia in order to accelerate and improve bone healing. The evolution of the case was demonstrated through radiographies.

Bone healing after a fracture occurs in three stages: inflammatory, reparative and remodelling. The inflammatory phase is the first and can last for several days. In this phase the initial hematoma is organised, cells are removed, and precursors cells are recruited (Roush, 2005). The reparative phase usually lasts about 6–10 weeks and is the time where the fracture gap is filled by material resembling bone. In this phase, proper bone healing occurs (Roush, 2005). The final phase is the remodelling phase, and the material resembling bone in the fracture gap is replaced by longitudinal haversian systems over time (Roush, 2005). Treatment for fractures aims to reestablish the anatomic position of the joint surfaces in relation to the proximal and distal bones in order to promote stability and allow the limb to reach early ambulation, with no impairments in gait (Roush, 2005; Glyde & Arnett, 2006). There is evidence that PBMT‐sMF may act in the first two stages of bone healing, modulating inflammatory process (Babuccu et al., 2014; Tim et al., 2016; Pretel et al., 2007) and enhancing reparative process (Pretel et al., 2007; Fazilat et al., 2014; de Almeida et al., 2014).

There are few clinical studies investigating the effects of PBMT or PBMT‐sMF on bone healing in the veterinary and human area. However, there is evidence from preclinical studies that PBMT helps vascular proliferation, modulates inflammatory process (Babuccu et al., 2014; Tim et al., 2016; Pretel et al., 2007) increases deposition of collagen in the osteoid matrix, osteoblast proliferation (Pretel et al., 2007; Fazilat et al., 2014; de Almeida et al., 2014) besides increasing biochemical properties of bone, expression of bone proteins and genes (Tim et al., 2016). These effects are able to enhance bone healing after a fracture by accelerating regeneration and remodelling of bone (Escudero et al., 2019).

The findings of this case report agree with the evidence mentioned above regarding the positive effects of PBMT on bone healing (Escudero et al., 2019). It was observed that 3 weeks after the fracture occurred, and after 18 days of treatment with PBMT‐sMF, the healing process of bone was advanced. Furthermore, 8 weeks after the fracture occurred there was no signs of any disruption in its continuity and fracture line, added to a closure of fracture gap.

Several fractures are proven difficult to manage such as distal radial fractures in small‐breed dogs, salter fractures of the distal ulnar physis of immature dogs, distal femoral fractures in immature dogs, and humeral fractures (Roush, 2005). In this case report, the dog had an immature skeleton, but the proximal tibia fracture is easier to manage. However, it is not possible to know if the bone healing would be adequate or if there could be some problem and, eventually, impairment of gait. In addition, the recommendation after surgery to repair fractures is activity restriction and controlled supervision (Roush, 2005). However, in this case, as the puppy was very active, damage to the external fixator or the plate could occur. In this way, both the owner and the vet were concerned about the fracture in the puppy. Therefore, faster healing could reduce the risk of nonunion or refracture and this could be achieved with the use of PBMT‐sMF.

One of the limitations of this case report is that the assessment of fracture healing was performed only by radiography. Furthermore, there were no other outcomes, other animals or a control group evaluated. Therefore, it is not possible to infer that the same results would or would not have been achieved if PBMT‐sMF had not been applied. Finally, a long‐term follow‐up was not carried out in order to observe the third bone healing stage, the remodelling phase. On the other hand, short‐ and medium‐term radiographs were taken, which allowed us to follow the first two stages of bone healing.

## 4 CONCLUSIONS

Although this case report has reported the use of PBMT‐sMF after a MIPO procedure in a puppy with complete transverse fracture in the right proximal tibia, randomised controlled trials are needed to affirm that PBMT‐sMF accelerates and improves bone healing after fracture. It is necessary to continue investigating the effects of PBMT and PBMT‐sMF on bone healing after fracture in both veterinary and human area.

## AUTHOR CONTRIBUTIONS

IMAC and AAM: conceptualisation. IMAC, AAM and DSJ: methodology. IMAC: writing‐ original draft preparation. IMAC and AAM: visualisation, investigation. DSJ: supervision. IMAC, AAM and DSJ: writing, reviewing and editing. All authors have read and agreed to the published version of the manuscript.

## FUNDING

This research did not receive any specific grant from funding agencies in the public, commercial, or not‐for‐profit sectors.

## CONFLICT OF INTEREST

Douglas Scott Johnson is an employee and shareholder of Multi Radiance Medical (Solon, OH, USA). The remaining authors have no conflicts of interests to declare.

### ETHICS STATEMENT

Not applicable because all the information derived from necessary clinical interventions.

*Patient consent statement*: For the procedures informed, written consent was obtained from the owncer of the animal.

### PEER REVIEW

The peer review history for this article is available at https://publons.com/publon/10.1002/vms3.1071.

## Data Availability

All data sets generated for this study are included in the article.

## References

[vms31071-bib-0001] Anders, J. J. , Lanzafame, R. J. , & Arany, P. R. (2015). Low‐level light/laser therapy versus photobiomodulation therapy. Photomedicine and Laser Surgery, 33, 183–184. 10.1089/pho.2015.9848 25844681 PMC4390214

[vms31071-bib-0002] Babuccu, C. , Keklikoğlu, N. , Baydoğan, M. , & Kaynar, A. (2014). Cumulative effect of low‐level laser therapy and low‐intensity pulsed ultrasound on bone repair in rats. International Journal of Oral and Maxillofacial Surgery, 43, 769–776. 10.1016/j.ijom.2013.12.002 24467933

[vms31071-bib-0003] de Almeida, A. L. , Medeiros, I. L. , Cunha, M. J. , Sbrana, M. C. , de Oliveira, P. G. , & Esper, L. A. (2014). The effect of low‐level laser on bone healing in critical size defects treated with or without autogenous bone graft: an experimental study in rat calvaria. Clinical Oral Implants Research, 25, 1131–1116. 10.1111/clr.12239 23919887

[vms31071-bib-0004] Dörtbudak, O. , Haas, R. , & Mailath‐Pokorny, G. (2002). Effect of low‐power laser irradiation on bony implant sites. Clinical Oral Implants Research, 13, 288–292. 10.1034/j.1600-0501.2002.130308.x 12010159

[vms31071-bib-0005] Draper, W. E. , Schubert, T. A. , Clemmons, R. M. , & Miles, S. A. (2012). Low‐level laser therapy reduces time to ambulation in dogs after hemilaminectomy: A preliminary study. Journal of Small Animal Practice, 53(8), 465–469. 10.1111/j.1748-5827.2012.01242.x 22783835

[vms31071-bib-0006] Drygas, K. A. , McClure, S. R. , Goring, R. L. , Pozzi, A. , Robertson, S. A. , & Wang, C. (2011). Effect of cold compression therapy on postoperative pain, swelling, range of motion, and lameness after tibial plateau leveling osteotomy in dogs. Journal of the American Veterinary Medical Association, 238(10), 1284–1291. 10.2460/javma.238.10.1284 21568773

[vms31071-bib-0007] Escudero, J. S. B. , Perez, M. G. B. , de Oliveira Rosso, M. P. , Buchaim, D. V. , Pomini, K. T. , Campos, L. M. G. , Audi, M. , & Buchaim, R. L. (2019). Photobiomodulation therapy (PBMT) in bone repair: A systematic review. Injury, 50, 1853–1867. 10.1016/j.injury.2019.09.031 31585673

[vms31071-bib-0008] Fazilat, F. , Ghoreishian, M. , Fekrazad, R. , Kalhori, K. A. , Khalili, S. D. , & Pinheiro, A. L. (2014). Cellular effect of low‐level laser therapy on the rate and quality of bone formation in mandibular distraction osteogenesis. Photomedicine and Laser Surgery, 32, 315–321. 10.1089/pho.2013.3559 24905927

[vms31071-bib-0009] Glyde, M. , & Arnett, R. (2006). Tibial fractures in the dog and cat: Options for management. Iris Veterinary Journal, 59, 290–295.

[vms31071-bib-0010] Gorse, M. J. (1998). Using external skeletal fixation for fractures of the radius and ulna and tíbia. Veterinary Medical, 93, 463–467.

[vms31071-bib-0011] Hoisang, S. , Kampa, N. , Seesupa, S. , & Jitpean, S. (2021). Assessment of wound area reduction on chronic wounds in dogs with photobiomodulation therapy: A randomized controlled clinical trial. Veterinaria World, 14(8), 2251–2259. doi: 10.14202/vetworld.2021.2251-2259 PMC8448658 34566346

[vms31071-bib-0012] Hudson, C. C. , Pozzi, A. , & Lewis, D. D. (2009). Minimally invasive plate osteosynthesis: Applications and techniques in dogs and cats. Veterinary and Comparative Orthopaedics and Traumatology, 22(3), 175–182. 10.3415/VCOT-08-06-0050 19448871

[vms31071-bib-0013] Impellizeri, J. A. , Tetrick, M. A. , & Muir, P. (2000). Effect of weight reduction on clinical signs of lameness in dogs with hip osteoarthritis. Journal of the American Veterinary Medical Association, 216(7), 1089–1091. 10.2460/javma.2000.216.1089 10754668

[vms31071-bib-0014] Johnson, A. L. , & Boone, E. G. (1993). Fractures of the tibia and fibula. In D. Slatter (Ed.), Textbook of small animal surgery (Vol. 2, 2nd edn., 1866–1876). Saunders, Philadelphia.

[vms31071-bib-0015] Johnson, A. L. , Houlton, J. E. F. , & Vannini, R. (2005). AO principles of fracture management in the dog and cat. New York: Thieme.

[vms31071-bib-0016] Karu, T. (1989). Laser biostimulation: a photobiological phenomenon. Journal of Photochemistry and Photobiology B Biology, 3(4), 638–640. 10.1016/1011-1344(89)80088-0 2507763

[vms31071-bib-0017] Looney, A. L. , Huntingford, J. L. , Blaeser, L. L. , & Mann, S. (2018). A randomized blind placebo‐controlled trial investigating the effects of photobiomodulation therapy (PBMT) on canine elbow osteoarthritis. Canadian Veterinary Journal, 59(9), 959–966.. PMC6091142 30197438

[vms31071-bib-0018] Palmer, R. H. (1999). Biological osteosynthesis. The Veterinary Clinics of North America. Small Animal Practice, 29(5), 1171–1185. vii. 10.1016/s0195-5616(99)50108-3 10503290

[vms31071-bib-0019] Pinheiro, A. L. , & Gerbi, M. E. (2006). Photoengineering of bone repair processes. Photomedicine and Laser Surgery, 24, 169–178. 10.1089/pho.2006.24.169 16706695

[vms31071-bib-0020] Plumb, D. C. (2005). Plumb's veterinary drug handbook. Stockholm, Wis.: Ames, Iowa: PhrmaVet; Distributed by Blackwell Pub.

[vms31071-bib-0021] Pozzi, A. , & Lewis, D. (2009). Surgical approaches for minimally invasive plate osteosynthesis in dogs. Veterinary and Comparative Orthopaedics and Traumatology, 22(4), 316–320. 10.3415/VCOT-08-10-0096 19597635

[vms31071-bib-0022] Pretel, H. , Lizarelli, R. F. , & Ramalho, L. T. (2007). Effect of low‐level laser therapy on bone repair: Histological study in rats. Lasers in Surgery and Medicine, 39, 788–796. 10.1002/lsm.20585 18081142

[vms31071-bib-0023] Rogatko, C. P. , Baltzer, W. I. , & Tennant, R. (2017). Preoperative low level laser therapy in dogs undergoing tibial plateau levelling osteotomy: A blinded, prospective, randomized clinical trial. Veterinary and Comparative Orthopaedics and Traumatology, 30(1), 46–53. 10.3415/VCOT-15-12-0198 27935005

[vms31071-bib-0024] Roush, J. K. (2005). Management of fractures in small animals. The Veterinary Clinics of North America. Small Animal Practice, 35, 1137–1154. vi. doi: 10.1016/j.cvsm.2005.06.001 16129136

[vms31071-bib-0025] Saygun, I. , Karacay, S. , Serdar, M. , Ural, A. U. , Sencimen, M. , & Kurtis, B. (2008). Effects of laser irradiation on the release of basic fibroblast growth factor (bFGF), insulin like growth factor‐1 (IGF‐1), and receptor of IGF‐1 (IGFBP3) from gingival fibroblastos. Lasers in Medical Science, 23, 211–215. 10.1007/s10103-007-0477-3 17619941

[vms31071-bib-0026] Schmokel, H. G. , Hurter, K. , & Schawalder, P. (2003). Percutaneous plating of tibial fractures in two dogs. Veterinary and Comparative Orthopaedics and Traumatology, 16, 191–195.

[vms31071-bib-0027] Schmökel, H. G. , Stein, S. , Radke, H. , Hurter, K. , & Schawalder, P. (2007). Treatment of tibial fractures with plates using minimally invasive percutaneous osteosynthesis in dogs and cats. Journal of Small Animal Practice, 48(3), 157–160. 10.1111/j.1748-5827.2006.00260.x 17355607

[vms31071-bib-0028] Schütz, M. , & Südkamp, N. P. (2003). Revolution in plate osteosynthesis: New internal fixator systems. Journal of Orthopaedic Science, 8(2), 252–258. 10.1007/s007760300044 12665968

[vms31071-bib-0029] Schwarz, G. (2005). Fractures of the tibial diaphysis. In: A.L. Johnson , J.E.F. Houlton , & R. Vannini (Eds.), AO principles of fracture management in the dog and cat (pp. 319–331). Davos (Switzerland): AO Publishing.

[vms31071-bib-0030] Taha, S. K. , El Fattah, S. A. , Said, E. , Abdel‐Hamid, M. A. , Nemat, A. H. , & El Shenawy, H. (2018). Effect of laser bio‐stimulation on mandibular distraction osteogenesis: An experimental study. Journal of Oral and Maxillofacial Surgery, 76(11), 2411–2421. 10.1016/j.joms.2018.04.030 29856939

[vms31071-bib-0031] Tim, C. R. , Bossini, P. S. , Kido, H. W. , Malavazi, I. , von Zeska Kress, M. R. , Carazzolle, M. F. , Parizotto, N. A. , & Rennó, A. C. (2016). Effects of low level laser therapy on inflammatory and angiogenic gene expression during the process of bone healing: A microarray analysis. Journal of Photochemistry and Photobiology B: Biology, 154, 8–15. 10.1016/j.jphotobiol.2015.10.028 26599085

[vms31071-bib-0032] Zaal, M. D. , & Hazewinkel, H. A. (1996). Classificatie van 202 tibiafracturen bij hond en kat [Classifications of 202 tibial fractures in dogs and cats]. Tijdschrift Voor Diergeneeskunde, 121, 218–223. 8669052

